# The painless ST-elevation myocardial infarction equivalent: a case report

**DOI:** 10.3325/cmj.2025.66.173

**Published:** 2025-04

**Authors:** Martin Medvid, Salome Glauser, Fabian Zürcher

**Affiliations:** 1 Emergency Center, SRO Langenthal, Langenthal, Switzerland; 2 Clinic for Cardiology, SRO Langenthal, Langenthal, Switzerland

## Abstract

This report presents the case of a 61-year-old patient who experienced sporadically occurring episodes of chest pain lasting approximately 15 minutes. The initial electrocardiogram (ECG) showed unspecific repolarization disturbances but no ST-elevation indicative of ST-elevation myocardial infarction (STEMI). However, upon closer examination, biphasic T waves were detected, suggestive of specific repolarization abnormalities. The conventional Wellens criteria were met, possibly indicating an etiopathogenetic correlation with the patient's complaints. Subsequent coronary angiography revealed a functional occlusion of the middle segment of the left anterior descending artery, which was treated by percutaneous transluminal coronary angioplasty/drug eluting stent. It also revealed a severely stenosed distal circumflex artery, indicating a two-vessel coronary disease. If we had used only conventional STEMI criteria, this patient would have certainly been missed. Therefore, when evaluating patients presenting with chest pain, it is imperative to consider non-occlusion infarction ECG abnormalities, known as STEMI equivalents. This case, moreover, highlights the importance of the non-officially proposed occlusion myocardial infarction (OMI)/non-OMI paradigm instead of the old STEMI/non-STEMI dichotomy.

In the age of highly specialized medicine with cardiac magnetic resonance imaging (MRI), stress echocardiography, and high-sensitivity troponin, the almost 150-year-old electrocardiogram (ECG) seems like diagnostics from a bygone era.

However, ECG persists as a painless, non-invasive, repeatable, and cost-effective examination procedure that can be performed almost anywhere. Therefore, it still continues to be – along with highly differentiated high-sensitivity cardiac troponin (hsTroponin) algorithms – the main criterion for the detection and management of myocardial ischemia for the differentiation between ST-elevation myocardial infarction (STEMI) and non-STEMI. Here, we report on a case where the failure to recognize subtle ECG changes, in the context of the patient's clinical presentation, could have resulted in a missed diagnosis, potentially leading to the patient’s death.

## CASE REPORT

### Medical history

A 61-year-old male patient presented at the emergency center of the SRO clinic, Langenthal, in September 2019 at night with a sporadic episode of chest pain lasting 15 minutes and ceasing spontaneously. Similar pain attacks had occurred frequently in the past two months and lasted up to one hour, accompanied by radiation into both arms, but not by shortness of breath. The patient did not notice any clear triggers such as physical stress, cold, food intake, or similar. At the moment of presentation, the patient was symptom-free.

His medical history included arterial hypertension and dyslipidemia – treated with a sartan and a statin – and a history of active smoking (cumulative 30 pack-years). His father had died at 50 years of age at his third myocardial infarction. The patient had never had a cardiac workup before.

### Status and clinical appearance

The patient presented in cardiopulmonary compensation, normotensive, with normal heart rate, afebrile, spontaneously well oxygenating, and in good general and slightly obese nutritional condition. Further examination was inconspicuous; no pathological heart sounds or pulmonological abnormalities were found.

**Figure 1:**
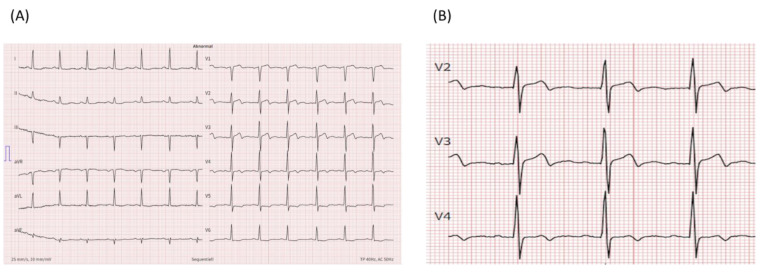
(**A**) Initial electrocardiogram (ECG) with a Wellens type-A morphology V2, V3, and possibly V4, preserved R progression and non-significant ST depression laterally (V5, V6, I, aVL), and a T wave flattening or maybe slight inversion, which we judged to be a non-specific repolarization disturbance. (**B**) Focused display: besides classical morphology of Wellens type A, we see how, in V2 and V3, the terminal T wave inversion is frequently only slightly emphasized. It forms a disproportionately small part of the whole width of the T wave; V4 appears more as a symmetrical “up-down” T wave.

The initial ECG (Figure 1) excluded a STEMI, displaying a normal sinus rhythm, regular intervals, and preserved R-progression. On closer examination, there were nonspecific Q-waves inferiorly (III, aVF), alongside precordial biphasic T waves, suggesting a repolarization abnormality. The timeline of the events and interventions is presented in Table 1.

### Diagnosis

**Figure 2:**
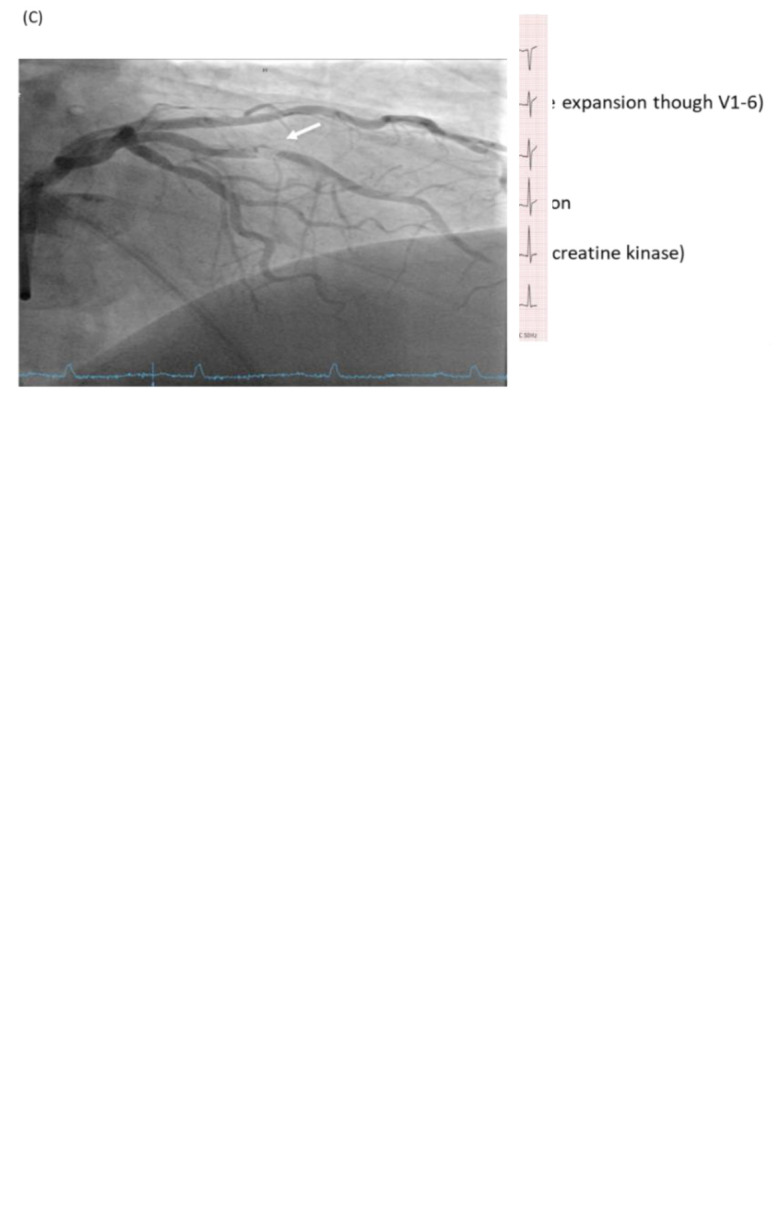
(**A**) The Wellens syndrome, diagnostic criteria according to Rhinehardt (1). (**B**) Progressive electrocardiogram (ECG) of our patient with borderline positive ST-elevation myocardial infarction (STEMI) criteria over the anterior wall. (**C**) The white arrow indicates the location of the coronary artery occlusion affecting the middle segment of the left anterior descending artery (right anterior oblique 44/cranial 26 projection).

The first recorded ECG showed a Wellens type-A T wave, ie, a biphasic “up-down” T wave (often interpreted as a terminal T wave inversion) in leads V2, V3, and V4, correlating with dynamic anterior wall hypoperfusion in the area of the left anterior descending (LAD) artery. Considering the patient's history, the absence of pain at admission, and the patient's cardiac risk profile, a possible etiopathogenetic explanation for his complaints was the Wellens syndrome. The conventional Wellens criteria (Figure 2A, B) were met. Since the Wellens sign was not primarily detected, laboratory results including cardiac enzymes were anticipated for the suspected diagnosis of non-STEMI. In the meantime, a new episode of pain occurred, and a second ECG was performed (Figure 2C), now showing most of conventional STEMI criteria.

Together with a typical clinical picture and clear ST-segment dynamics, a coronary problem was almost certain. After administration of acetylsalicylic acid and unfractionated heparin, the patient was transferred directly to our reference hospital with cardiac catheterization laboratory for coronarography. During the transfer, the first hsTroponin T measurement showed an increased value of 58 ng/L (cutoff >14 ng/L). In the catheterization laboratory (Figure 2D), a functional occlusion of the mid-LAD was treated by percutaneous transluminal coronary angioplasty/drug eluting stent. Moreover, a severely stenosed distal circumflex artery was found, indicating a two-vessel coronary disease. After a successful placement of the stent, the patient was transferred back to our hospital with a favorable outcome and discharged after two days for ambulatory cardiac rehabilitation.

## DISCUSSION

Here, we reported on a case where the failure to recognize subtle ECG changes, in the context of the patient's clinical presentation, could have resulted in a missed diagnosis, potentially leading to the patient´s death. The interdisciplinary debriefing of the case was dominated by the dilemma of STEMI vs NSTEMI. However, the real question to pose in potentially ischemic patients should be occlusion vs non-occlusion.

STEMI criteria are neither specific nor sensitive. In the DIFOCCULT study, 25%-30% of all occluded coronary arteries presented as NSTEMI and thus remained unnoticed until delayed coronarography (2). Martie et al, in their ST-segment deviation analysis of 116 patients by cardiac contrast MRI, showed a sensitivity of the current STEMI criteria of 50%. After co-assessment of ST-depression as an addition to non-significant ST-elevation, the sensitivity increased to 84% with only a minimal loss of specificity (3).

In a prospective study of 504 patients, 18% had insufficient ST-elevations on ECG, although occlusion/subocclusion was present; 91% of them required percutaneous coronary intervention (4).

The largest meta-analysis examining cardiovascular endpoints including mortality in over 40 000 NSTEMI patients showed that 25% NSTEMI patients had a total coronary occlusion with an increased risk for a worse endpoint in contrast to 75% NSTEMI patients without occlusion (5).

The extent of occlusion correlates poorly with the millimeters of ST elevation. For over 20 years, physicians in emergency medicine and cardiologists have tried to introduce the occlusive myocardial infarction (OMI) and non-occlusive myocardial infarction (NOMI) “paradigm shift” (2,6).

Currently, the term “STEMI equivalent” is used when patients show coronary occlusions requiring immediate cardiac catheterization despite not meeting the STEMI criteria. Currently, the best described STEMI equivalents are the following:

1) The De Winters T waves: dominant precordial T waves coming directly from an ST-depression, associated with a proximal- to mid-LAD occlusion.

2) The diagonal branch (D1) occlusion, associated with isolated aVL and V2 ST-elevation.

3) The isolated posterior infarction, characterized by horizontal ST-depression at the anterior precordial leads.

4) Mainstem ischemia with a diffuse ST-depression and isolated ST-elevation in aVR.

5) Left-bundle branch block (native or artificial with pacemaker) with positive Smith-modified Sgarbossa and/or Barcelona criteria (7-9).

6) The Wellens syndrome or Wellens sign, characterized by precordial dynamic and terminal T wave inversions (10).

In our patient, the first ECG showed the previously described precordial T wave changes. The differential diagnosis of T wave abnormalities is one of the broadest in electrocardiography. It poses an essential difference whether the T wave abnormality is diffuse (as seen in electrolyte disturbances, habitus, pulmonary disease, hyperventilation, hypothermia, intoxication, etc) or localized in coronary distribution (ie, most likely ischemic). Especially when it comes to the localized T wave abnormalities, the dynamic changes may help in assigning the ischemic etiology of the repolarization disturbance.

Biphasic T waves occur frequently as expressions of hypokalemia. The diffuse “down-up” T waves can take gargantuan forms when developing rapidly (“Himalayan” T waves). The terminally positive component of the biphasic T wave in hypokalemia can be explained by a superimposed U-wave. The QTc time significantly increases, and the patient often needs magnesium in addition to potassium as a precaution.

However, a reverse configuration of biphasic T waves (“up-down”) may indicate a STEMI equivalent (Wellens type A). These waves are typically not diffuse but distributed according to the coronary supply area. In the classic Wellens syndrome (10,11), it is the supply area of the proximal- to mid-LAD. The etiopathogenesis of the syndrome remains unclear. It is postulated that the terminally negative component of the biphasic Wellens type-A T wave exists due to the reperfusion of the adjacent ischemic component. QTc time is minimally prolonged, and the patient usually requires care according to STEMI guidelines, despite the absence of typical pain.

Wellens type-B T waves are found in patients with complete spontaneous reperfusion. In this case, deep symmetric T wave inversions are eminent, commonly pointing to the supply area of the proximal- to-mid LAD. Paradoxically, re-occlusion leads to the disappearance of the Wellens sign and hence to completely physiological ECGs. However, patients usually report anginal symptoms. Reperfusion occurs completely in each case in classic Wellens syndrome and hence, completely preserved R waves remain over the anterior wall. This back-and-forth between occlusion and reperfusion can continue over hours, days, or even weeks.

However, as in our patient’s case, repetitive reperfusion as a compensation mechanism of the coronary artery is not guaranteed; persistent occlusion may, therefore, occur at any time. In this situation, the pain will typically persist, electrocardiographically there will be a progression of the positive component of the T wave with eventual development of a hyperacute T wave and ST-elevations, and thus a STEMI situation. The recognition of such a Wellens syndrome may help to prevent future transmural myocardial infarction.

In conclusion, emergency physicians have to bear in mind that STEMI criteria are neither specific nor sensitive for coronary occlusion. Furthermore, in addition to a correctly assessed new-onset left-bundle branch block, other STEMI equivalents must be interpreted correctly. Finally, even in the modern era of medicine, ECG is still a fast, inexpensive, and indispensable tool in the diagnosis and treatment of patients in the emergency department. A close examination of ECG is particularly important if STEMI criteria are not met. If we applied only conventional STEMI criteria, our patient could have been easily missed. This case, moreover, highlights the necessity of the non-officially proposed OMI-NOMI paradigm, in contrast to the old STEMI-NSTEMI dichotomy.

**Table 1:** The timeline of events and interventions

Time	Event
23:00	Presentation to the emergency department, triaged by nursing staff: currently pain free, with a history of intermittent thoracic pain.
23:20	Medical history and clinical examination, initial electrocardiogram (ECG).
00:10	A recurrent chest pain episode, serial ECG shows dynamic changes.
00:20	Decision to perform invasive coronary angiography, administration of acetylsalicyc acid and unfractionated heparin.
00:35	Transfer to the central hospital for coronary angiography.
00:40	First laboratory results: slightly elevated high-sensitivity troponin T.
01:15	Coronary angiography performed, admission to the cardiology intermediate care unit.
